# Food Safety Concerns: *Anisakis* spp. in Ready-to-Eat Fish from the Greek Market

**DOI:** 10.3390/pathogens14100981

**Published:** 2025-09-27

**Authors:** Evangelia N. Papapostolou, Serafeim C. Chaintoutis, Panagiota Gousia, Aggeliki Karpouza, Melania Kachrimanidou, Anastasia Diakou

**Affiliations:** 1 Department of Food Testing and Research Laboratories of Thessaloniki, Hellenic Food Authority (EFET), 57001 Thessaloniki, Greece; epapapostolou@efet.gr (E.N.P.); pgousia@efet.gr (P.G.); akarpouza@efet.gr (A.K.); 2 Diagnostic Laboratory, School of Veterinary Medicine, Faculty of Health Sciences, Aristotle University of Thessaloniki, 54627 Thessaloniki, Greece; schainto@vet.auth.gr; 3 Department of Microbiology, Medical School, Aristotle University of Thessaloniki, 54124 Thessaloniki, Greece; 4 Laboratory of Parasitology and Parasitic Diseases, School of Veterinary Medicine, Faculty of Health Sciences, Aristotle University of Thessaloniki, 54124 Thessaloniki, Greece

**Keywords:** Anisakidae, anisakidosis, *Anisakis pegreffii*, *Anisakis simplex* sensu stricto, fish products, Greece

## Abstract

Nematode parasites of the family Anisakidae can cause human disease when ingested, at the third larval stage (L3), through infected fish or fish products. While human infection is often asymptomatic, in some cases it may present with symptoms ranging from mild to severe gastrointestinal disorders, skin rash, itching, or even anaphylaxis. Ingestion of destroyed L3 (e.g., following processing, sanitisation, or cooking) may still trigger allergic reactions. In the present study, the occurrence of Anisakidae L3 in ready-to-eat (RTE) fish products was investigated. A total of 108 RTE fish products, representing eight species and five processing methods, were examined using (a) macroscopic inspection, (b) artificial digestion (AD), and (c) molecular analysis of both the AD material (to detect non-visible parasite fragments) and the larvae collected by the first two methods. Nematodes morphologically identified as Anisakidae L3 were detected in 32 of the 108 samples (29.62%). Molecular analysis of the AD product did not reveal additional positive samples and confirmed the identification of the isolated larvae as *Anisakis simplex* sensu stricto and *Anisakis pegreffii*. Given the public health importance of anisakidosis, the findings of this study provide valuable insight into the prevalence of contamination in RTE fish products currently available on the Greek market.

## 1. Introduction

The nematodes of the family Anisakidae are among the primary zoonotic parasites that infect marine species, causing human anisakidosis. The genera of clinical interest include *Anisakis* (*Anisakis simplex* sensu stricto (s.s.) and *Anisakis pegreffii*), *Pseudoterranova* (*Pseudoterranova azarazi*, *Pseudoterranova cattani*, *Pseudoterranova decipiens*, and *Pseudoterranova krabbei*), and, more rarely, the genus *Contracaecum* (*Contracaecum osculatum*) [1]. Dolphins and whales, in the case of *Anisakis*, pinnipeds, in the case of *Pseudoterranova*, and fish-eating birds like pelicans and penguins, in the case of Contracaecum are the definitive hosts of these parasites. Small crustaceans serve as intermediate hosts, while cephalopods and fish act as paratenic hosts, carrying the infective third-stage larvae (L3). Humans can be become infected as paratenic and dead-end hosts (i.e., they do not contribute to the completion of the parasite’s life cycle) when consuming raw, undercooked, or inadequately frozen infected fish [1,2,3].

The global distribution and abundance of Anisakidae are attributed to their adaptability to a wide range of hosts and environmental conditions. In wild-caught fish, infection is common, although prevalence varies depending on the fish species and fishing area. Numerous studies have examined the occurrence of these parasites in commercially consumed fish [4,5,6]. In Greece, similar research has revealed a significant presence of Anisakidae in the Aegean Sea [7,8].

Humans are at high risk of infection when consuming raw, undercooked, or inadequately processed fishery products containing the L3 [9]. Anisakidosis may present with different clinical syndromes, depending on larval migration within the body (i.e., gastric, intestinal, or extra-gastrointestinal anisakidosis), as well as the potential onset of an allergic reaction (allergic anisakidosis) [10,11]. Clinical signs appear within a few hours after infection, including mild to severe gastrointestinal disorders and symptoms similar to those seen in gastric ulcers [2]. In certain cases, the larvae penetrate the intestinal wall, leading to extra-gastrointestinal inflammatory lesions that may cause severe abdominal pain resembling acute appendicitis and requiring surgical intervention. They may also induce granulomatous nodules in the peritoneum, which can be misdiagnosed as neoplastic lesions [10,12,13]. Allergic reactions, such as rashes, dermatitis, urticaria, rhinoconjunctivitis, and asthma, may occur after consuming either live or dead larvae [11,14,15,16]. In addition to consumers, fishermen and fishmongers who come into contact with infected fish and cephalopods are at risk of sensitisation and allergic reactions from ingesting or handling infected fish and cephalopods, or even asthma from inhaling antigens from these parasites [17]. Moreover, allergic reactions may occur after consuming meat of farmed poultry that has been fed contaminated seafood [18].

Anisakidosis is a serious emerging food-borne disease that is now spreading to all continents due to changing eating habits, international cuisine, population movements, and international trade, posing a risk to public health and economic challenges in fisheries and food safety [19]. Between 2001 and 2023, *Anisakis* spp. accounted for 73.6% of all parasitic infection notifications in the European Union’s Rapid Alert System for Food and Feed (RASFF) database, underscoring real-world implications of the problem [20]. The first report of anisakiasis due to *Anisakis* sp. in Greece, caused by the consumption of raw fish, serves as a critical example [21]. Furthermore, epidemiological data suggest that anisakidosis may be an underdiagnosed disease [2,12].

Food business operators (FBOs) producing fishery products must comply with legislation requirements to ensure food safety. In the European Union, Regulation (EC) 853/2004 supplemented by Regulation (EC) 2074/2005 and amended by Regulation (EC) 1276/2011 establishes health standards for producing safe fishery products including mandatory treatment to inactivate viable parasites and the visual inspection of catches to prevent contaminated fish from entering the market. This Regulation includes specific requirements such as the mandatory treatment of fish to eliminate viable parasites that pose health risks to consumers. Effective methods include freezing or heating at specific temperatures and time periods [22,23,24].

Despite these preventive measures, international literature indicates that Anisakidae L3 have been detected in processed foods widely distributed on the international market. Live larvae have been found in ready-to-eat (RTE) mackerel [25], marinated anchovies lacking further heat treatment [26], and smoked herring [27]. The efficacy of marination, smoking, and salting in inactivating Anisakidae larvae is influenced by parameters such as brine salinity, pH, and exposure duration [28,29,30,31]. Nevertheless, multiple studies have demonstrated that common marination and cold-smoking protocols are insufficient to ensure complete larval inactivation. As a result, regulatory authorities mandate preventive freezing for fishery products intended for raw or lightly processed consumption, as in the case of RTE products [32]. This study aimed to investigate the prevalence, species composition, and intensity of Anisakidae nematodes infections in RTE fish products available on the Greek market. Given that the risk of infection in farmed fish is considered negligible [33], products derived from such fish were excluded from the analysis. The findings contribute to mapping the contamination of RTE fish products in Greece and underscore a potential foodborne source of morbidity.

## 2. Materials and Methods

### 2.1. Sampling

A total of 108 samples of RTE fish products, labelled as originating from wild-caught fish, were randomly purchased between September and December 2024 from six supermarket chains in the city of Thessaloniki, all of which have branches across Greece. The production facilities were primarily located in central and northern Greece and had received official approval from the relevant authorities.

The sampled products included smoked, salted, and marinated fish of two types: one consisting of whole fish and the other consisting of fish fillets, preserved in oil or water. The fishery products included herring (*Clupea harengus*), mackerel (*Scomber scombrus*), Atlantic chub mackerel (*Scomber colias*), anchovy (*Engraulis encrasicolus*/*Engraulis ringens*), sardine (*Sardina pilchardus*), pollock (*Pollachius virens*), albacore (*Thunnus alalunga*), and Skipjack tuna (*Katsuwonus pelamis*). The products’ classification and the location of catches are provided in Table 1.

After collecting, the products were transported to the laboratory. Labelling information was recorded, and the samples were stored at a controlled temperature of 4 °C. Testing was initiated within a 48 h timeframe. Epidemiological indices were applied according to Bush et al. 1997 [34].

### 2.2. Artificial Digestion and Macroscopic Inspection

The methodology for artificial digestion (AD) adhered to the guidelines established by the European Union Reference Laboratory for Parasites (EURLP) [35], which is grounded in ISO 23036-2021 [36]. The method was applied as follows.

For sample preparation, fillets were drained of the preserving medium (oil or water). Salted fish were rinsed under tap water for 10 s to remove surface salt. The skin was then removed, the fish eviscerated, and the muscle tissue separated. Throughout the process, macroscopic inspection of both the discharged liquid (oil, brine or rinse water) and the fish was carried out to detect and collect parasites when present.

For each test, samples of either 50 or 100 g were used per product, depending on the product type. For larger fish species, such as herring and mackerel, 100 g of muscle tissue was sampled from the abdominal region, where larvae are more likely to be present according to ISO 23036-2021.

Visceral organs of non-eviscerated smoked herring were removed and examined separately. For smaller fish, the tests included 1 to 7 fish from the same batch, ensuring that the total size of each sample reached 100 g. The fillets were tested individually by package, in 50 g portions.

The following ingredient ratios were used to prepare the digestion liquid: In 2 L of tap water 10.8 mL of hydrochloric acid (HCl, 37%) was added. Ten grams of pepsin powder (1:10.000 NF; Opopharma, Handels GmbH, Hamburg, Germany) were incorporated into the mixture. The prepared sample was placed in a 2 L glass beaker containing the digestion liquid. A sample-to-digestive fluid ratio of 1:20 was maintained during the AD process. The beaker was covered with aluminium foil to maintain a stable temperature of 40–42 °C and to minimize evaporation. The temperature of the fluid was monitored regularly with a thermometer, and the stirring was adjusted to produce a deep vortex without splashing. The sample was digested for 30 min.

At the end of the procedure, the AD product was passed through a sieve, and the detected parasites were collected, counted and stored in saline solution at −20 °C for further examination.

### 2.3. Morphological Identification

The collected larvae were examined without further processing under the microscope (×100, ×400 magnification) for morphological identification at the family level [37,38,39].

### 2.4. Molecular Examination

Molecular analysis was performed on two types of material: (i) the sediments obtained from the AD (approximately 2 mL) to detect larvae potentially destroyed by production procedures or the AD process, and (ii) one larva per positive sample. The larvae were dissected, and DNA extraction was performed from their midsection. A previously described methodology for DNA extraction [40] was followed with minor modifications [41]. This process involved mixing and homogenising the sediment or larvae in DNA lysis buffer, followed by the application of a phenol/chloroform-based procedure, and subsequent binding to silica columns.

Molecular detection and identification involved PCR-based amplification of a 628 bp fragment of the mitochondrial cytochrome oxidase II (*cox2*) subunit gene, using the previously described primers 211F and 210R [42]. The QIAgen HotStarTaq DNA Polymerase was used in reaction mixtures, and amplification was performed in a SimpliAmp Thermal Cycler (Thermo Fisher Scientific, Waltham, MA, USA). A total of 10 μL from each PCR product were analysed by electrophoresis on a 1.5% agarose gel (110 V for 100 min), followed by staining with GelRed Nucleic Acid Gel Stain (Biotium Inc., Fremont, CA, USA) and visualisation under UV, to verify amplicon lengths. The PCR products were subsequently purified using the NucleoSpin Gel and PCR Clean-up kit (Macherey-Nagel, Düren, Germany). Sanger sequencing was performed bidirectionally, utilising the amplification primers. Forward and reverse sequencing reads were assembled using MEGA 11 software [43], and primer sequences were trimmed. Assembled sequences were analysed via BLAST for identification.

## 3. Results

**Figure 1:**
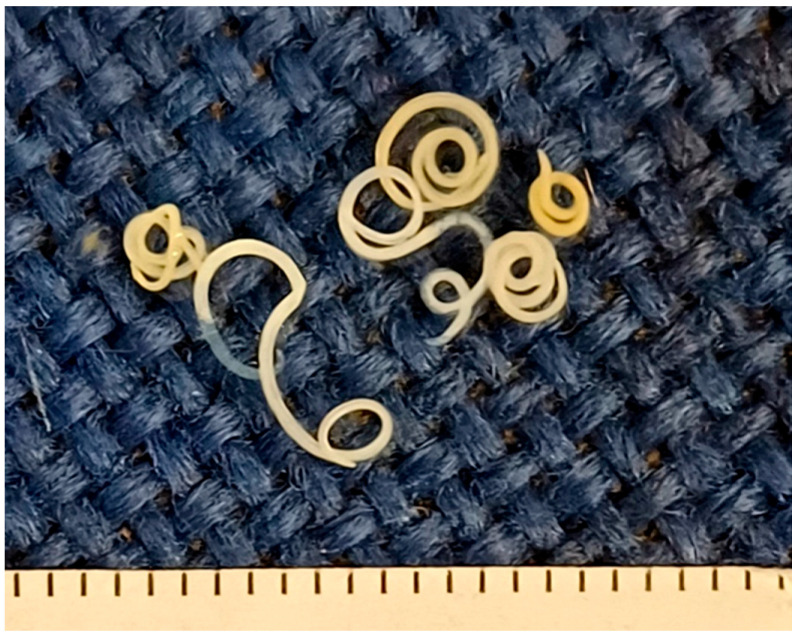
Nematodes isolated from ready-to-eat fish products from the Greek market. The scale (bottom) indicates millimetres.

**Figure 2:**
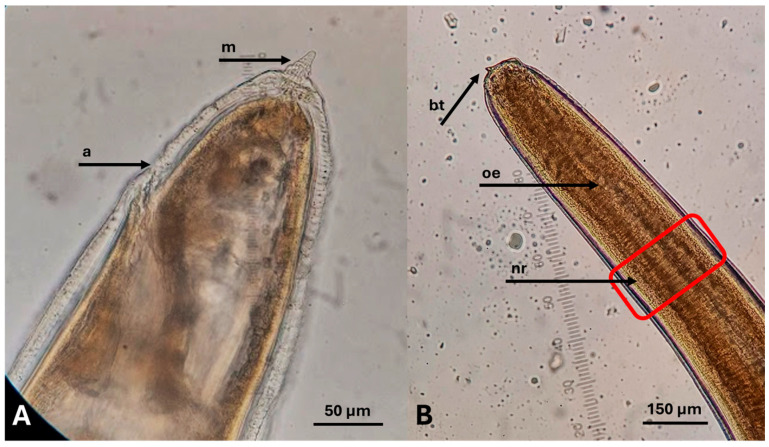
Morphology of Anisakidae third-stage larvae, found in ready-to-eat fish products from the Greek market. (**A**) Posterior end. a: anus, m: mucron; (**B**) Anterior end. bt: boring tooth, oe: oesophagus, nr: nerve ring.

White to pale yellow coiled nematodes were detected in 32 of the 108 (29.62%) examined samples of RTE fish products (Figure 1). In all positive samples, parasites were detected only after AD, whereas none were observed in the fish or the discharged liquid during visual inspection during the preparation process. None of the parasites detected showed any sign of viability during observation and manipulation. Based on their morphological and morphometric characteristics (Figure 2), all isolated larvae were identified as belonging to the family Anisakidae.

The highest prevalence of infection was found in smoked whole herring (91.6%), followed by salted whole Atlantic chub mackerel (47.0%), smoked whole mackerel (46.6%), smoked mackerel fillet in oil (18.2%), marinated anchovy fillet in oil (12.5%), smoked herring fillet in oil (11.1%), and salted whole anchovies (10.0%). No larvae were found in salted whole sardines in oil, salted pollock fillet in oil, smoked albacore fillet in oil, or smoked Skipjack tuna fillet in water (Table 2).

The highest mean intensity of infection (number of larvae per sample) was found in salted whole Atlantic chub mackerel (7.5), followed by smoked whole herring (5.63), smoked whole mackerel (2.42), smoked mackerel fillet in oil (1.5), marinated anchovies fillet in oil (1.5), smoked herring fillet in oil, (1) and salted whole anchovies (1). No mobility of the larvae was observed during macroscopic examination after AD.

The molecular analysis of partial *cox2* gene sequences obtained from the larvae confirmed the morphological identification and assigned all processed larvae as members of the *Anisakis* genus, showing high identity (≥99%) with *Anisakis* spp. reference sequences. The sequences from larvae recovered from Atlantic chub mackerel and anchovies displayed the highest nucleotide sequence identity (99.6–100%) to *A. pegreffii* reference sequences (e.g., acc. MF960821.1, KY418058). In contrast, the sequences from larvae recovered from herring and mackerel showed the highest nucleotide sequence identity (99.3–100%) to *A. simplex* s.s. reference sequences (e.g., acc. PQ126420.1, KT852492.1) (Table 3).

## 4. Discussion

This is the first survey to investigate the presence of Anisakidae in RTE fish products from the Greek market. The prevalence of infection ranged from 10.0% to 91.6%, depending on the product, demonstrating that these parasites represent a common challenge in food production. The significance of Anisakidae parasites mainly lies in their contribution to foodborne disease. Although these parasites have been recognised in fish since the 13th century, they were first documented in humans in Greenland in 1876 [44]. The first notable case of symptoms associated with the parasite was reported in 1950, when Dr. Straub in the Netherlands isolated a nematode from an eosinophilic granuloma in the small intestine of a patient suffering from acute abdominal pain [44].

Currently, thousands of cases of anisakidosis are reported worldwide each year, with Japan documenting over 19,000 cases annually and Spain reporting more than 7000 [45]. The first case in Greece was reported in 2024 in a young male with a history of regularly consuming raw fish [21]. It involved an invasive *Anisakis* sp. infection mimicking peritoneal malignancy, which led to hemicolectomy and omentectomy. The diagnosis of anisakiasis (infection by parasites of the genus *Anisakis*) was subsequently confirmed through histopathology and molecular identification [21].

The broad range of definitive, intermediate, and paratenic hosts of Anisakidae parasites, together with their adaptability to diverse environmental conditions, underlies their worldwide distribution. Fishing regions, including the Mediterranean, Japan, and the Atlantic Ocean, frequently report the occurrence of these parasites [1]. Commercially caught fish often contain Anisakidae larvae, which vary in prevalence depending on the fish species and catch area. Near the Canary Islands, five different species of *Anisakis*, including the zoonotic *A. simplex* (s.s.) and *A. pegreffii*, were found in frigate tunas (*Auxis thazard*), European hake (*Merluccius merluccius*), Atlantic chub mackerel and mackerel [4]. The infectious stage (L3) of *A. pegreffii*, *Anisakis typica*, and *Anisakis ziphidarum* was isolated from scorpionfish (*Scorpaena scrofa*) caught in the Aegean Sea [46] and from anchovies in the Western Mediterranean and Adriatic Sea [5,26,47]. The Atlantic herring is one of the marine fish species with a high prevalence and intensity of *A. simplex* infection, which has led to the parasite’s common name, the “herring worm” [6]. In the present study, molecular identification based on partial *cox2* gene sequencing indicated that all isolated larvae belonged to the *Anisakis* genus, showing the highest sequence identity with reference sequences of either *A. simplex* (s.s.) or *A. pegreffii*. Recent research has also reported the occurrence of hybrid genotypes between *A. simplex* (s.s.) and *A. pegreffii* [48,49]. A limitation of the present study was that only a single genetic locus was analysed. The application of multilocus approaches is recommended for more robust species-level discrimination and for the detection of potential hybrid genotypes, similar to what has already been proposed [50].

Studies conducted in fresh fish in Greece have demonstrated significant parasite circulation in the Aegean Sea. Infected species include the European cod (*Merlangius merlangus*), Atlantic chub mackerel, and mackerel [7]. Research in the marine area around Lesvos Island reported an exceptionally high prevalence of infection (98.8%) in grey horse mackerel (*Trachurus trachurus*) [8].

Post-mortem migration of larvae into the edible flesh of fish represents a significant concern for food safety. Studies on *A. pegreffii* confirm significant post-mortem migration into fish musculature after 5 and 3 days at storage temperatures of 0 and 4 °C, respectively, triggered by biochemical factors such as pH and accumulation of biogenic amines [51]. Migration of *A. pegreffii* larvae from the viscera to the fillets was positively associated with both storage temperature (5 °C and 7 °C) and storage time (24, 48, and 72 h), with significantly higher worm burdens in the fillets and a simultaneous decrease in larval counts in the viscera as a result [5].

Conversely, no migration of *A. simplex* s.s. was observed in herring and mackerel stored < 2 °C, even after 48 h. However, this was not the case in blue whiting (*Micromesistius poutassou*), where at the same conditions, significant migration of *A. simplex* s.s. took place, showing that host species likely plays a crucial role in this phenomenon [52,53]. Overall, although maintaining storage temperatures below 2 °C prevents migration to an extend [52], early evisceration is strongly advocated in scientific literature [54] as a best practice to reduce migration risk, though it is not a legally enforceable requirement.

The influence of viscera on the occurrence and intensity of infection, depending also on the parasite species is evident in the present results; samples containing viscera were more frequently infected and exhibited higher infection intensities with *A. simplex* s.s. (Table 2 and Table 3). Specifically, smoked whole herring showed an infection prevalence of 91.6% and a mean intensity of 5.63, whereas smoked herring fillets showed markedly lower values of 11.1% and 1, respectively. A comparable pattern was observed for smoked whole mackerel and smoked mackerel fillets. In contrast, anchovies infected with *A. pegreffii* displayed no substantial differences in infection prevalence or intensity regardless of the presence of viscera. This observation may indicate that *A. pegreffii* has a greater propensity to migrate from the viscera to the muscles than *A. simplex* s.s., although further evidence is needed to confirm this.

Migration of the larvae from the viscera to the muscles may not always be the case in infected fillets; It has been shown in different species of salmon from Alaska returning from the open sea that *A. simplex* s.s. larvae are more abundant in the musculature than in the viscera immediately after catch, while interestingly, no migration of the larvae is observed from the viscera into the flesh during storage on ice or in refrigerated seawater for 24 h [53]. *Intra vitam* migration is a natural phenomenon that has been documented for both *A. pegrefii* and *A. simplex*, of which intensity depends on both host and parasite species and biology [5,52].

Studies examining the presence of Anisakidae in RTE fish products remain limited. In a survey conducted in Turkey by AD on 205 samples of various local RTE products from different fish species, no larvae were detected [55]. The examination by AD of 107 samples of RTE salted or marinated anchovies from Italian supermarkets revealed a high infection prevalence of 54.2% [56]. The authors emphasise that, although the risk of anisakidosis from dead parasites is negligible, the risk of allergic reactions remains significant. Furthermore, the presence of parasites in these products may pose a major market problem, as products with visible infection are rejected by consumers [56]. Notably, in the present study, no parasites were detected by naked eye before AD, despite the heavy infection in some cases, indicating that infection can remain unnoticed by the consumer. In a relatively recent survey, RTE anchovy (n = 45) and sardine (n = 45) fillets were collected from different markets in Italy and Spain [57]. In 17 anchovy and 13 sardine products, larvae were detected and identified as *A. pegreffii* (30%) and *A. simplex* (70%). The fillets were salted, in-oil preserved, and all the larvae were non-viable [57].

Interestingly, a study of RTE mackerel in Japan detected a notably high proportion of live larvae using tissue compression and parasite extraction [25]. These products consisted of fresh fish fillets with bones and viscera removed but had not undergone any processing, such as freezing, smoking, salting, or marinating. The lack of processing likely explains the high prevalence of live parasites in these RTE products. It has been demonstrated that AD may underrate the viability of L3 in frozen fish products [58]. The absence of live L3 in RTE products after AD should be interpreted with caution, as the procedure may deactivate the parasites [58]. In the present study, the larvae detected showed no signs of viability, which could be attributed either to the effect of processing methods (smoking, salting, or marinating) which may provide a safer alternative than freezing [32], particularly when freezing does not comply with established regulations [22], or to the effect of the AD per se.

Fishery products companies must comply with the legislation’s requirements for safe food production. Regulation (EC) 853/2004 supplemented by Regulation (EC) 2074/2005 [23] and amended by Regulation (EC)1276/2011 [24] enforce the treatment to kill viable parasites in fishery products for human consumption and mandates the visual inspection of catches to remove contaminated products from the market establishing health standards for producing safe fishery products [19]. It dictates the processing of catches to eliminate viable parasites that pose health risks to consumers. Specifically, catches must be frozen at −20 °C for at least 24 h or at −35 °C for at least 15 h, or the centre of mass must be heated above 60 °C for at least 1 min. Salting has been proposed as an alternative method of inactivating Anisakidae larvae [32]. The EFSA guidelines specify that ≥9% NaCl for ≥6 weeks, 10–20% NaCl for ≥4 weeks, or >20% NaCl for ≥3 weeks are effective for inactivating Anisakidae larvae in fish products [59]. Experimental data corroborate these findings [60]: 15% NaCl achieves complete larval inactivation within 7 days, 18.6% NaCl (via brining and dry salting) in baccalà achieves inactivation in 15 days, and 21% NaCl in anchovies also resulted in complete inactivation over 15 days [30]. Traditional marination (e.g., herring) with lower salt concentrations (≈9%) requires 5–6 weeks for complete inactivation, while reducing salt notably increases survival times [28]. These studies robustly support the assertion that low-salt or lightly salted fish (i.e., <10% NaCl) may not inactivate larvae, whereas higher concentrations (around 10% or above) combined with extended exposure are required.

Processing technologies can reduce the presence of larvae in products reaching the market but visual inspection remains essential and should be performed by well-trained people [57]. However, it is noteworthy that in the present study no parasites were detected during visual inspection of the products; in all cases, parasites were detected only after AD, highlighting the utility of this method when investigating the prevalence of infection in fish and fish products. The efficacy of alternative methods, such as irradiation, high hydrostatic pressure, and drying, has yet to be fully documented. Traditional methods such as marinating and cold smoking are not always sufficient to inactivate larvae. For example, it has been shown that although dry-salting is effective in destroying *Anisakis* spp. [31] the effect of marinating procedures depends on factors such as salt concentration, acidity and time [28,29,30]. Thus, further research and information to validate these and other techniques, including the use of essential oils, is warranted [33].

Nonetheless, it is important to consider the presence of even dead Anisakidae parasites in fish products, as they may trigger allergic reactions in sensitive individuals [14]. To minimise the presence of larvae in the edible parts of fish, operators employ various removal methods, including early gutting, filleting, and trimming, carried out manually or mechanically. These techniques are generally highly effective in ensuring parasite-free fish products [33]. The consumption of fish products prepared from aquaculture fish could be a safer option for *Anisakis*-sensitised patients, provided that the feed of these fish is free of *Anisakis* antigens [61].

## 5. Conclusions

The present survey demonstrates that *A. simplex* s.s. and *A. pegreffii* are present in RTE fish products available on the Greek market. The overall prevalence of infection is similar to that found in other EU markets of the Mediterranean [56,57], but greatly depends on the fish species and the presence of viscera in the product (whole fish or fillets only). The prevalence observed herein highlights the magnitude of the challenge in producing parasite-free RTE fish products. The presence of *Anisakidae* larvae in RTE products has important implications for the seafood industry. Contaminated products can result in recalls, financial losses, and reduced consumer confidence, potentially affecting market demand. Beyond compliance with existing EU regulations (e.g., freezing requirements and visual inspection), the industry can reduce risk by implementing additional preventive measures, such as rapid evisceration after catch, strict temperature control during storage and transport, and adoption of enhanced inspection or detection technologies. Regular staff training and HACCP plans that specifically address parasitic hazards can further improve safety. These steps not only mitigate the risk of infection but also help maintain consumer trust in seafood products, ultimately benefiting public health and market stability.

## Figures and Tables

**Table 1:** Type of fish product, number of products examined, packaging, and fishing area.

Type of Product	No. of Products	Packaging Net Weight (on Average)	Fishing Area
Smoked whole herring	12	12 × 200 g	FAO 27
Smoked herring fillet in oil.	9	9 × 110 g	FAO 27
Smoked whole mackerel	15	15 × 300 g	FAO 27
Smoked mackerel fillet in oil	11	11 × 100 g	FAO 27
Salted whole Atlantic chub mackerel	17	17 × 100 g	FAO 37
Salted whole anchovies	10	10 × 100 g	FAO 37
Marinated anchovies fillet in oil	16	16 × 50 g	FAO 37/FAO 87
Salted whole sardines in oil	5	5 × 70 g	FAO 37
Salted pollock fillet in oil	5	5 × 80 g	FAO 27
Smoked albacore fillet in oil	3	3 × 100 g	FAO 37/FAO 47
Smoked Skipjack tuna fillet in water	5	5 × 80 g	FAO 71

FAO 27: Atlantic, Northeast; FAO 37: Mediterranean & Black Sea; FAO 47: Atlantic, Southeast; FAO 71: Pacific, Western Central FAO 87: Pacific, Southeast.

**Table 2:** Ready-to-eat fish products found infected with Anisakidae larvae and infection intensity per sample.

Type of Product	No. of Samples	Prevalence (%)	No. of Retained Larvae	Mean Intensity *	No. of Larvae per Sample
Smoked whole herring	12	11 (91.6)	62	5.63	9–15
Smoked herring fillet in oil	9	1 (11.1)	1	1	1
Smoked whole mackerel	15	7 (46.6)	17	2.42	1–6
Smoked mackerel fillet in oil	11	2 (18.2)	3	1.5	1–2
Salted whole Atlantic chub mackerel	17	8 (47.0)	60	7.5	5–11
Salted whole anchovies	10	1 (10.0)	1	1	1
Marinated anchovies fillet in oil	16	2 (12.5)	3	1.5	1–2
Salted whole sardines in oil	5	0 (0)	0	na	na
Salted pollock fillet in oil	5	0 (0)	0	na	na
Smoked albacore fillet in oil	3	0 (0)	0	na	na
Smoked Skipjack tuna fillet in water	5	0 (0)	0	na	na
Total	108	32 (29.62)	147	na	na

* mean number of larvae per sample, na: non-applicable.

**Table 3:** Results of molecular analysis performed on the AD sediments and isolated larvae.

Type of Product	No. of Samples	Molecular Analysis (AD Sediments)	No. of Positive Samples by AD (Presence of L3)	Molecular Analysis (Larvae)
Smoked whole herring	12	Neg.	11 + 2 *	*A. simplex* s.s.
Smoked herring fillet in oil	9	Neg.	1	*A. simplex* s.s.
Smoked whole mackerel	15	Neg.	7	*A. simplex s.s.*
Smoked mackerel fillet in oil	11	Neg.	2	*A. simplex s.s.*
Salted whole Atlantic chub mackerel	17	Neg.	8	*A. pegreffii*
Salted whole anchovies	10	Neg.	1	*A. pegreffii*
Marinated anchovies fillet in oil	16	Neg.	2	*A. pegreffii*
Salted whole sardines in oil	5	Neg.	0	N/A **
Salted pollock fillet in oil	5	Neg.	0	N/A **
Smoked albacore fillet in oil	3	Neg.	0	N/A **
Smoked Skipjack tuna fillet in water	5	Neg.	0	N/A **

* Parasites detected in viscera of 11 samples; in 2 of these, also in muscle; ** N/A: not applied.

## Data Availability

Data is contained within the article. No voucher specimens were deposited in a formal collection. The material has been preserved and is available upon request. Metadata linking each specimen to its host, product type, and collection site is maintained to allow verification of morphological and molecular identifications.

## Abbreviations

The following abbreviations are used in this manuscript:

AD	artificial digestion
RTE	ready-to-eat
FBOs	food business operators
HACCP	Hazard Analysis and Critical Control Points
